# COVID-19, Intimate Partner Violence, and Communication Ecologies

**DOI:** 10.1177/0002764221992826

**Published:** 2021-02-06

**Authors:** Clare E. B. Cannon, Regardt Ferreira, Frederick Buttell, Jennifer First

**Affiliations:** 1 UC Davis, Davis, CA, USA; 2 University of the Free State, Bloemfontein, South Africa; 3 Tulane University, New Orleans, LA, USA; 4 University of Tennessee–Knoxville, Knoxville, TN, USA

**Keywords:** IPV, communication ecology, COVID-19

## Abstract

The purpose of this research is to identify important predictors, related to the ongoing COVID-19 pandemic, of intimate partner violence (IPV) and to provide insight into communication ecologies that can address IPV in disaster contexts. This study uses a cross-sectional design, with purposive snowball sampling, for primary survey data collected over 10 weeks starting the first week in April 2020. A total of 374 adults participated in the study. Logistic binary regression was used to identify key predictors among sociodemographic characteristics, stress related to COVID-19, and perceived stress of group membership for those who reported IPV experiences. A *t* test was used to statistically differentiate between IPV-reporters and non-IPV reporters based on perceived stress measured by the Perceived Stress Scale. Results indicated that respondents who reported renting, lost income due to COVID-19, and increased nutritional stress were all more likely to belong to the IPV-reporters group. These findings provide insight into additional stressors related to the ongoing pandemic, such as stress due to income loss, nutritional stress, and renting, and their likelihood of increasing IPV victimization. Taken together, these results indicate that additional communication resources are needed for those affected by IPV. Additional findings and implications are further discussed.

The global outbreak of the novel coronavirus disease 2019 (COVID-19) has spread to every country across the world, infecting millions and killing hundreds of thousands of people (John Hopkins University, 2020), and warranting a pandemic declaration by the World Health Organization (WHO; 2020). In its wake, COVID-19 has exacerbated mental health problems, such as depression and anxiety (e.g., Mazza et al., 2020; Smith et al., 2020; Wang et al., 2020). It has created a global economic recession, while continuing to threaten public health. Even before the pandemic led to a lock-down around the world, the WHO estimated that 35% of women globally experienced physical or sexual violence from an intimate partner over the course of their lives (WHO, 2017). Although COVID-related lock downs, when adequately followed, have successfully slowed the transmission of the virus, they have most likely contributed to a separate epidemic of physical violence and mental health issues (Holmes et al., 2020; Horesh & Brown, 2020).

Key public health strategies like social distancing, social isolation, and stay-at-home orders have slowed the spread of COVID-19, but have created ideal conditions for intimate partner violence (IPV) to flourish (Holmes et al., 2020). For example, these same strategies increase the amount of time that women have to spend home alone with their abusive partners furthering their social isolation (WHO, 2020). IPV is defined here as physical, emotional, psychological, or economic abuse and stalking or sexual harm by a current or former partner or spouse (Center for Disease Control and Prevention, 2020). The United Nations Population Fund estimates a 20% increase in IPV globally due to quarantines and lock downs (Stanley, 2020). Moreover, external relational stressors, such as unemployment brought on by COVID-19, increase opportunities for relational conflict (Capaldi et al., 2012). Compounding these stressors, women fleeing abusive partners have no place to go with shelters also locked down (Brooks, 2020; Mock, 2020; Taub, 2020).

Previous research into disasters and IPV has found that disasters tend to increase both the frequency and intensity of IPV (Buttell & Carney, 2009; Ferreira et al., 2018; Lauve-Moon & Ferreira, 2017). Such research may prove insightful in understanding how well COVID-19-related stressors predict experiences of IPV amid the COVID-19 pandemic. Moreover, communication ecologies, or a conceptual model for understanding and analyzing different forms and strategies of communication, may prove useful in furthering our understanding of the relationships among disaster, IPV, and public health responses to multiple crises (i.e., COVID-19, IPV, and economic hardships). Research into disaster, IPV, and communication ecologies may provide guidance for developing effective response and recovery strategies in the wake of cascading adverse effects due to the pandemic.

## Intimate Partner Violence, Disasters, and COVID-19

IPV is a pernicious and preventable social and public health problem, where approximately 25% of women and 10% of men in the United States experience some form of IPV in their lifetime (Center for Disease Control and Prevention, 2020). According to U.S. crime reports, approximately 16% of all homicide victims are killed by an intimate partner (Cooper & Smith, 2011; Lutgendorf, 2019; Stöckl et. al, 2013; Velopulos et al., 2019). IPV is also associated with other adverse health outcomes such as cardiovascular, reproductive, musculoskeletal, and nervous system conditions (Black, 2011; Campbell, 2002; Carbone-López et al., 2006; Sugg, 2015). The prevalence and severity of IPV has likely been exacerbated by the ongoing disaster of COVID-19 (Holmes et al., 2020; Wanqing, 2020), while research on IPV in disaster contexts continues to be understudied. Of the scant literature available, research suggests that IPV increases in prevalence and severity postdisaster (First et al., 2017; Harville et al., 2018; Molyneaux et al., 2019; Norris, 2005, 2014; Parkinson & Zara, 2013; Rao, 2020). Previous disaster research suggests that disaster specific stressors may increase IPV victimization. For instance, research found hurricane-related stressors such as if respondents had been displaced from their home, lost sentimental possessions, or experienced shortage of food or water due to the hurricane, predicted an increase in IPV victimization (Schumacher et al., 2010). Research into technological disasters has similarly suggested that those affected by such disasters are twice as likely to experience both physical and emotional IPV than those not affected by disaster (Lauve-Moon & Ferreira, 2017).

Moreover, previous studies suggest that both experiences of disasters and IPV are associated with greater perceived stress (Ferreira et al., 2018; Frasier et al., 2004; Schwartz et al., 2015). Perceived stress is understood here as the degree to which a person perceives a threat from a stressor and how capable they feel in behaviorally and cognitively adapting to it (Caplan, 1981; Lazarus & Folkman, 1984). Similarly, IPV research has found, using the Perceived Stress Scale (PSS; Cohen, 1994), that increased stress has been associated with higher levels of depression (Rodriguez et al., 2010), that perceived stress mediates relationships between IPV and cardiovascular disease (Wright et al., 2019), and that IPV victims report higher levels of perceived stress, psychological distress, and somatic complaints (Bonomi et al., 2006; Dillon et al., 2013; Frasier et al., 2004; Williams et. al 2020).

COVID-19 can be understood as a disaster with similarities to other kinds of disasters associated with natural and technological hazards along with important differences (Buttell & Ferreira, 2020; Ferreira et al., 2020). Similar to other kinds of disasters, recent research on the ongoing pandemic suggests high rates of mental health problems among general populations due to the COVID-19 pandemic, including the fear of contagion as well as stress related to social distancing and economic hardship (Kira et al., 2020; Mazza et al., 2020; Smith et al., 2020; Wang et al., 2020). Recent research also suggests several key differences between COVID-19 and other kinds of disasters. For instance, Ferreira et al. (2020) found that unlike other kinds of disasters, survey respondents who anticipated needing greater reliance on neighbors and family evidenced less resilience than those who indicated they perceived needing less reliance on others.

Additionally, external relational stressors like unemployment and housing instability either caused by or exacerbated by COVID-19 increase opportunities for relational conflict (Capaldi et al., 2012). This, in turn, might increase the risk for more frequent and intense abuse in families already experiencing IPV prior to COVID-19. A problem unique to the situation caused by COVID-19 is that there is no place for IPV victims to go to escape the abuse. They may call a hotline number and receive psychological assistance over the phone, but police are being strongly encouraged to not make arrests for anything other than felonies. Also due to COVID-19, IPV shelters are not open or being staffed for those fleeing abusive relationships (Brooks, 2020; Elinson & Chapman, 2020; Mock, 2020; Taub, 2020). Recent media coverage further suggests that IPV is increasing globally, with reports from Wuhan Province, China that IPV rates were 3 times higher in February 2020 than the same time period the previous year (Wanqing, 2020), with similar reports in Spain, Italy, and Brazil (Kelly, 2020). Because of the similarities and differences of COVID-19 to other disasters, more research is necessary to understand how disaster response and recovery strategies can be leveraged in response to COVID-19 and reduce IPV victimization.

## Communication Ecologies

A communication ecology is a conceptual model used to analyze and visualize relationships among social interactions, discourses, and communication materials that individuals depend on to achieve a goal (Broad et al., 2013; Shah et al., 2017; Turner et al., 2010). Communication ecologies have been defined as “a network of communication resource relations constructed by individuals in pursuit of a goal and in context of their communication environment” (Ball-Rokeach et al., 2012, p. 4). Recently, communication ecologies have been applied in the context of disasters to construct a framework toward understanding how communication resources are utilized across multiple systems (e.g., micro, meso, macro) to cope with challenges occurring before, during, and after disasters (Houston, 2012; Perreault et al., 2014; Spialek & Houston, 2018). For example, disaster communication ecologies can encompass various systems and sectors such as individuals, community organizations, media (e.g., traditional and social), disaster warning systems, and governmental organizations (Houston, Hawthorne, et al., 2015). Within these various systems and sectors, disaster communication resources fulfill important functions such as receiving information about a disaster (Perreault et al., 2014), creating and sustaining social support (Houston, Spialek, et al., 2015), and engaging in disaster coping (Spialek & Houston, 2019; Spialek et al., 2016). Disaster communication ecologies, therefore, provide an important role in understanding how various communicative systems, resources, and relationships assist in coping with the challenges before, during, and after disaster.

In the context of IPV and disaster, scholars have highlighted the importance of communication systems and resources to cultivate and promote safety for IPV victims before, during, and after disaster. For example, Buttell and Carney (2009) examined the capacity of the New Orleans Police Department to respond to IPV calls before and after Hurricane Katrina. Their study highlights the centrality of communication resources being available between law enforcement and individuals to help ameliorate IPV effects during disaster. Likewise, First et al. (2017) outline an IPV and disaster framework for IPV professionals to cultivate various capacities that promote IPV victim safety and well-being before, during, and after a disaster. Each of the capacities in the IPV and disaster framework include several components of communication. For instance, capacities include communicative processes such as disseminating disaster and IPV risk information, education, and planning; coordinating disaster and IPV responses; enhancing social support; and constructing survivor narratives (First et al., 2017). While scholars have highlighted the important role of communication resources to cope with disaster and IPV (Buttell & Carney, 2009; First et al., 2017; Lauve-Moon & Ferreira, 2017), IPV has yet to be included within disaster communication ecology scholarship. Victims of IPV face compounding stressors and needs during disasters and research into the structure of IPV communication ecologies would provide guidance for developing effective IPV response and recovery strategies in communities experiencing disaster.

## Purpose of the Study

The overall purpose of this study is to determine levels of perceived stress related to the COVID 19/Coronavirus Pandemic among non-IPV and IPV respondents who experienced the first 10 weeks of the stay at home orders/lockdown during the COVID-19 pandemic and the role of family, friends, neighbors, and government and nongovernment resources in coping with IPV and COVID-19. The guiding research question was to investigate how much of the variance in experiencing IPV can be explained by individual experiences related to the COVID-19 pandemic. More specifically, the purpose of this study is threefold and aims to (a) investigate the role of perceived stress, individual-level stressors, and perceived reliance on resources experienced during the COVID-19 outbreak; (b) present findings from a study active during the COVID-19 pandemic; and (c) add to the literature on how research can better support communication to ameliorate IPV effects during disaster.

## Method

This study utilizes a cross-sectional design. Data for the study were collected over a 2-month period from an online survey launched the first week of April, 2020. The study was approved by the Tulane University Socio-Behavioral Institutional Review Board. The self-administered online survey was distributed through one of the authors’ personal social media accounts (e.g., Facebook, Instagram, and LinkedIn) and advertised on the Tulane University School of Social Work’s social media outlets and website for a period of 2 months. The main inclusion criteria for the online survey required participants to be older than 18 years and have direct access to the survey link. Exclusion criteria included those who were younger than 18 years. The survey focus was on participants’ (a) previous disaster experience, (b) perceived stress (i.e., PSS), (c) current situation as it relates to COVID-19, (d) experienced IPV, and (e) personal and household demographics. The online *Qualtrics* survey took approximately 10 minutes to complete.

### Participants

The study sample consisted of individuals having access to the online Qualtrics survey link. Participants were recruited for participation in the study through a mixture of purposive snow-ball sampling with one study author requesting and encouraging participants to share the survey link on their own personal social media accounts, as well as having the survey link displayed on the school’s home page and advertised in media outlets for the school. The sample for this study includes 374 adults who completed the online survey. Data analysis was conducted using SPSS 26.

### Measures

#### Outcome Variable

##### Intimate partner violence exposure

The study outcome variable was a binary variable assessing whether an individual experienced IPV (referent).

#### Predictor Variables

##### Perceived Stress Scale

The PSS is regarded as a classic stress assessment instrument and shows a correlation with a variety of health-related measures that include stress measures, self-reported health and health services measures, health behavior measures, smoking status, and health-seeking behaviors (Cohen, 1988, 1994). Scores on the PSS can range from 0 to 40, with higher values on the PSS indicating greater perceived stress. Scores ranging from 0 to 13 are considered low, 14 to 26 are considered moderate, and 27 to 40 are considered as high. The PSS has been shown to have excellent psychometric properties across a wide range of studies investigating stress (e.g., Lee, 2012; Roberti et al., 2006).

#### COVID-19 Experiences

The study included several questions related to exposure to COVID-19. The following predictor variables and response categories were included (a) has the COVID-19/Coronavirus lead to you losing your job (1 = *yes*; 0 = *no*); (b); has the COVID-19/Coronavirus lead to you losing income (1 = *yes*; 0 = *no*); (c) What is your residential status? (1 = *renter*; 0 = *homeowner*); (d) How often would you say you are worried or stressed about having enough money to pay your rent/mortgage? (1 = *always*, 2 = *usually*, 3= *sometimes*, 4 = *rarely*, 5 = *never*); (e) How often would you say you are worried or stressed about having enough money to buy nutritious meals? (1 = *always*, 2 = *usually*, 3= *sometimes*, 4 = *rarely*, 5 = *never*); (f) do you think you will need help and cooperation from others (e.g., family, friends, or neighbors) to recover from the impact of COVID-19/Coronavirus? (1 = *very little help*, 2 = *little help*, 3 = *neither very little help nor very much help*, 4 = *much help*, 5 = *very much help*); (g) do you think you will need help and cooperation from others (e.g., government or nongovernmental organizations) to recover from the impact of COVID-19/Coronavirus? (1 = *very little help*, 2 = *little help*, 3 = *neither very little help nor very much help*, 4 = *much help*, 5 = *very much help*); (h) how many days have you spent in “stay at home/lockdown.”

## Results

### Descriptive Statistics of the Total Sample

The sample of 374 participants had a mean age of 47.01 years (*SD* = 14.67) and was composed of 74.6% women (*n* = 279) and 25.4% men (*n* = 95). The majority of the sample identified as White (86.1%; *n* = 322). Regarding relationship status, the majority of participants were in a relationship, 55.9% (*n* = 209). The majority of the sample were employed, with 63.4% (*n* = 237) reporting being employed at the time of study participation. Regarding education, only 1.3% of respondents had less than a high school diploma (*n* = 5), while 3.5% had a high school diploma or GED (*n* = 13), followed by 12.8% (*n* = 48) with some college, 4.5% (*n* = 17) held an associate’s degree, 25.7% held a bachelor’s degree (*n* = 96), and the majority of the sample held a graduate degree 52.1% (*n* = 195). The majority of the sample spoke English at home 71.1% (*n* = 268). Nearly two-thirds of the sample reported owning their home (63.9%; n=239). Of the total sample, 10.4% (*n* = 39) reported having experienced IPV. Of respondents, 22.9% (*n* = 86) expressed having stress related to nutrition access. As it relates to mortgage and rent stress, roughly one third (33.6%; *n* = 126) of respondents expressed stress related to being able to meet mortgage or rent obligations.

### Intimate Partner Violence Survivor Demographics

The subsample of 39 participants who experienced IPV had a mean age of 46.97 years (*SD* = 14.5), and 74.4% identified as women (*n* = 29) and 25.6% as men (*n* = 10). The majority of IPV survivors identified as White 84.6% (*n* = 33). Roughly half of IPV-reporter were employed (55.1%, *n* = 20). Regarding education, one third of IPV survivors had a graduate degree (35.9%; *n* = 14). The majority of IPV-reporters spoke English at home (66.7%; *n* = 26). Nearly three-quarters (74.4%, *n* = 29) reported owning their home. Half of the IPV-reporters group expressed experiencing stress as it relates to nutrition access 48.7% (*n* = 19). Regarding mortgage and rent stress, 56.4% (*n* = 22) of participants expressed stress related to being able to meet mortgage or rent obligations.

Table 1 provides a detailed description of the demographic characteristics for the total sample and the IPV-reporter group.

**Table 1.** Demographic Characteristics of the Sample.

*Characteristic*	*Participants (N = 374)*		
	Non-IPV reporters, *n* = 335	IPV reporters, *n* = 39	Total, *N* = 374
*Age (in years)*	47.01 (*SD* = 14.7)	46.97 (*SD* = 14.5)	47.01 (*SD* = 14.6)
*Gender*			
*Male*	25.4 (85)	25.6 (10)	25.4 (95)
*Female*	74.6 (250)	74.4 (29)	74.6 (279)
*Race*			
*White*	86.3 (289)	84.6 (33)	86.1 (322)
*Minority*	13.7 (46)	15.4 (6)	13.9 (52)
*Relationship*			
*In a relationship*	55.5 (186)	59.0 (23)	55.9 (209)
*Single*	44.5 (149)	41 (16)	44.1 (165)
*Employment*			
*Employed*	64.8 (217)	51.3 (20)	63.4 (237)
*Unemployed*	35.2 (118)	48.7 (19)	36.6 (137)
*Education*			
*Less than 12 years/No HS diploma*	1.5 (5)	-	1.3 (5)
*HS diploma/GED*	3.0 (10)	7.7 (3)	3.5 (13)
*Some college*	12.2 (41)	17.9 (7)	12.8 (48)
*Associate degree*	4.5 (15)	5.1 (2)	4.5 (17)
*Bachelor degree*	24.8 (83)	33.3 (13)	25.7 (96)
*Graduate degree*	54.0 (181)	35.9 (14)	52.1 (195)
*English second language*			
*English*	72.2 (242)	66.7 (26)	71.7 (268)
*English second language*	27.2 (91)	33.3 (13)	28.3 (106)
*Housing status*			
*Own house*	62.7 (210)	74.4 (29)	63.9 (239)
*Rent*	37.3 (125)	25.6 (10)	36.1 (135)
*Stress*			
*Nutrition stress*			
*Yes*	20.0 (67)	48.7 (19)	22.9 (86)
*No*	80.0 (268)	51.3 (20)	77.1 (288)
*Rent/mortgage stress*			
*Yes*	31.0 (104)	56.4 (22)	33.6 (126)
*No*	69.0 (231)	43.6 (17)	66.4 (238)
*Perceived Stress Scale*			
*Perceived stress mean score*	16.02 (SD = 5.99)	19.33 (*SD* = 7.75)	16.37 (*SD* = 6.27)
*Help and cooperation from others (e.g., family, friends, or neighbors)*			
*Very little help*	37.3 (125)	25.6 (10)	36.1 (135)
*Little help*	27.8 (93)	20.5 (8)	27.0 (101)
*Neither very little help nor very much*	18.8 (63)	25.6 (10)	19.5 (73)
*Help*	9.6 (32)	20.5 (8)	10.7 (40)
*Much help*	3.0 (10)	5.1 (2)	3.2 (12)
*Very much help*	3.6 (120	2.6 (1)	3.5 (13)
*Don’t know/NA*			
*Help and cooperation from others (e.g., government or nongovernmental organizations)*			
*Very little help*	29.6 (99)	28.2 (11)	29.4 (110)
*Little help*	28.7 (96)	15.4 (6)	27.3 (102)
*Neither very little help nor very much*	17.9 (60)	20.5 (8)	18.2 (68)
*Help*	14.0 (47)	15.4 (6)	14.2 (53)
*Much help*	5.4 (18)	15.4 (6)	6.4 (24)
*Very much help*	4.2 (14)	5.1 (2)	4.3 (16)
*Don’t know/NA*			

*Note*. IPV = intimate partner violence.

### Analysis Strategy: Logistic Regression and Independent *t* Test

In order to investigate how much of the variance in experiences of IPV can be explained by perceived stress and personal experiences related to the COVID-19 pandemic, one binary logistic regression model was performed along with one independent *t* test analysis and two Wilcoxon-Mann–Whitney U tests. The regression model focused on experiences related to COVID-19 as predictors of experiences of IPV group membership and a *t* test tested whether there was a statistically significant difference between the IPV-experienced group and non-IPV experienced group with respect to perceived stress (i.e., total PSS score). To test communication ecology insights elaborated above, we used two Wilcoxon–Mann–Whitney U tests to investigate whether the IPV-experiences group rated the need for communication resources from specifically (a) family, friends, neighbors, and (b) government and nongovernment sources to recover from the impact of COVID-19 higher than the non-IPV experiences group.

### Logistic Regression Model: COVID-19 Experiences Predicting IPV Group Membership

A logistic regression was performed to investigate how well COVID-19 experiences (i.e., has COVID-19 lead to you losing your job; has COVID-19 lead to you losing income, renter, mortgage/rent stress, nutritional stress, and do you think you will need help and cooperation from others) predicts group membership based on IPV experiences. The model consisted of six predictors and allowed for simultaneous entry of all the independent variables.^1^ All assumptions of logistic regression were met. The odds ratios of the logistic regression model are presented in Table 2.

**Table 2.** Logistic Regression Analysis of IPV and COVID-19 Experiences.

	**Β**	***SE***	**Wald**	**Exp. (Β)**	**95% CI for Exp. (Β)**	
					LL	UL
**Job loss due to COVID-19**	0.275	0.409	0.452	1.1317	0.590	2.937
**Income loss due to COVID-19**	−0.743	0.377	3.881	0.476^*^	0.227	0.996
**Renters**	−0.833	0.419	3.954	0.435^*^	0.191	0.988
**Mortgage/rent stress**	0.275	0.479	0.330	1.136	0.515	3.365
**Nutritional stress**	1.166	0.466	6.267	3.208^*^	1.288	7.991
**Anticipated need for help from others**	0.316	.437	0.524	1.372	0.583	3.228
**Constant**	−1.902	1.148	2.744	0.149		

*Note. n* = 355. Degrees of freedom = 6. IPV = intimate partner violence; CI = confidence interval; LL = lower limit CI; UL = upper limit CI; *SE* = standard error.

* *p* < .05. ^**^p < .01. ^***^p < .001.

A test of the full model against a constant-only model was statistically significant (χ^2^ = 24.22, degrees of freedom [*df*] = 6, *p* = .001). The model *R*² indicates that the model accounted for 13.3% of the total variance. Prediction success for the cases used in the development of the model were high, with an overall success rate of 89.9%. This suggests that the set of predictors successfully discriminates between group membership based on experiences of IPV.

Three of the predictor variables, loss of income due to COVID-19 (Wald χ^2^ = 3.882, *df* = 1, *p* = .049, 95% CI [0.227, 0.996]), renter status (Wald χ^2^ = 3.954, *df* = 1, *p* = .047, 95% CI [0.191, 0.988]), and nutritional stress (Wald χ^2^ = 6.267, *df* = 1, *p* = .012, 95% CI [1.288, 7.991]) were statistically significant predictors of group membership based on IPV experiences. Based on the model, respondents who reported income loss due to COVID-19 were 47.2% more likely than those who did not lose income to report IPV-experience. Similarly, renters were 43.5% more likely than homeowners to belong to the IPV-experiences group. Respondents who reported nutritional stress were 3.2 times more likely to belong in the IPV-reporters group than respondents who did not report nutritional stress.

### Independent *t* test: Perceived Stress Group Differences

An independent sample *t* test was performed to determine if there was a difference between the non-IPV (did not experience IPV) and experienced IPV subsamples on the PSS. The mean PSS score for the non-IPV group was 16.02 (*SD* = 5.99) and for the experienced IPV group was 19.33 (*SD* = 7.75), indicating that the experienced IPV had higher levels of perceived stress compared with the non-IPV group. This difference was significant, *t*_(365)_ = −3.148, *p* = .002) with a small effect size (Cohen’s *d* = .4779). Results are presented in Table 3.

**Table 3.** Perceived Stress Differences Between Non-IPV and IPV Group.

Perceived Stress Scale	*n*	*M*	*SD*	*t*	*df*	*p*	Cohen’s *d*
Non-IPV^**^	328	16.02	5.99	−3.148	365	.002	.4779
**IPV**	39	19.33	7.75				

*Note. n* = 355. IPV = intimate partner violence; *df* = degrees of freedom.

* *p* < .05. ^**^p < .01. ^***^p < .001.

### Mann–Whitney Test: Perceived Resource Use Group Differences

A Wilcoxon–Mann–Whitney U test was performed to determine if those in the IPV experiences group rated a need for help and cooperation from family, friends, and neighbors to recover from the impact of COVID-19 higher than those in the non-IPV group. The mean rank for the IPV experiences group was 220.85 and for the non-IPV experiences group was 183.62, indicating that the IPV experiences group rated their need for help from others greater than the non-IPV experiences group. This difference is significant (U = 5232; *p* = .03). A second Wilcoxon-Mann-Whitney U test was performed to determine if those in the IPV experiences group rated a need for help and cooperation from government and nongovernment resources to recover from the impact of COVID-19 higher than those in the non-IPV group. The results were not significant. Results are presented in Table 4.

**Table 4.** Differences in Anticipated Need for Help and Cooperation.

Variable	*n*	Mean rank
**Reliance on family, friends, and neighbors**		
Non-IPV group	335	183.62
IPV group	39	19.33
Test statistics		
Mann–Whitney U	5232.00	
Wilcoxon W	61512.00	
Z	−2.12	
*p*	.03	
**Reliance on government and nongovernment resources**		
Non-IPV group	334	184.29
IPV group	39	210.19
Test statistics		
Mann–Whitney U	5608.5	
Wilcoxon W	61553.5	
Z	−1.46	
*p*	0.14	

*Note*. IPV = intimate partner violence.

## Discussion

### COVID-19 Stressors and IPV

The results of this study are important because they represent one of the first assessments of the relationships among the COVID-19 pandemic, IPV, and stress. This study found that although only 10% of the sample reported experiencing IPV during the pandemic, the people that had experienced IPV reported more stress globally than the segment of the sample that had not experienced IPV. Furthermore, the results of the *t* test indicate that as perceived stress increases, as measured by the PSS, participants are more likely to end up in the IPV subgroup. Importantly, these data do not suggest causality and there is no way to determine if IPV was present in those relationships prior to the pandemic. What the data do suggest, however, is that experiencing IPV is related to reporting more exposure to stress.

The results of the logistic regression equation help flesh out the relationship between stress exposure and experiencing IPV in a little more detail. That model suggests that as respondents were more likely to be renters, suffer a loss of income and be worried about adequate nutrition, they were more likely to experience IPV. This finding makes intuitive sense and is consistent with the extant literature on IPV and external stressors (e.g., Barton & Bryant, 2016; Capaldi et al., 2012; DeMaris et al., 2003; Neal & Edwards, 2017; Schwab-Reese et al., 2016). In brief, as people find themselves in a more tenuous financial situation due to COVID-19, there are more things to worry about and subsequently argue about. In many instances, that type of situation leads to an occasion for IPV. In our sample’s case, as people lost their jobs and suffered financial losses, they also likely increased their worry about eviction. This conclusion makes sense, as homeowners have been far more likely to receive accommodations from lenders who want to avoid another housing collapse like the one in 2008 (Goodman & Magder, 2020). However, many landlords still want their rent and the media has covered the issue of eviction moratoriums and the end of those protections (e.g., Goldstein, 2020). Consequently, throughout the pandemic many renters have feared they will be forced to leave when they can no longer pay rent. In fact, in a recent analysis of the relationship between concerns about housing and loss of income during the years following the great recession in 2008, Schneider et al. (2016) found that unemployment and economic hardship at the individual household level were positively related to abusive behavior. In essence, as people suffer from a lack of income and worry about paying their rent, there are more opportunities for relational conflict as uncertainly and anticipatory anxiety either exacerbate existing IPV or create the first occasion of IPV in a relationship.

The data generated for this study was collected during a 2-month period of the early pandemic, April and May 2020. It is notable that even that early in the pandemic, respondents were reporting a moderate level of stress. Apart from the timing of the data collection, the overall sample was White (86.1%), women (74.6%), employed (63.4%), and homeowners (63.9%) with either a college or graduate education (77.8%). Almost all of these attributes serve as protective factors in resilience models and yet the total sample reported experiencing moderate levels of stress at the beginning of the pandemic. One can only speculate on what the level of reported stress might be now, more than 6 months into the pandemic. Of particular concern for this study was the finding that the subset of the sample who reported experiencing IPV were significantly more stressed at the time of study participation than the non-IPV group. Although not captured in the data for this study, it seems reasonable to conclude that the overall level of stress experienced by this sample has increased dramatically since the time of study participation and that this increase has had a disproportionate negative impact of the subsample that experienced IPV.

### Communication Ecologies, Disasters, COVID-19, and IPV

The findings from this study have important implications for expanding communication ecology research related to IPV and disasters. The findings suggest that in pandemic disasters, the efforts to corral and contain the virus might inadvertently contribute to a dramatic rise in IPV. Therefore, to address the increased risk of IPV during a pandemic, IPV risk information, education, and planning should be foregrounded in conversations about public safety precautions in a pandemic (Bradley et al., 2020; Moreira & da Costa, 2020). For instance, in our survey, we used two questions to measure perceived resource needs from family and friends and a second question regarding government and nongovernment resources. Of the subsample that did not report IPV experiences, approximately 12% reported they anticipated needing help or much help from friends, families, or neighbors, while 25% of those in the IPV subsample reported the same. This is more than a twofold increase in terms of perceived reliance on others for help during a pandemic for those who are experiencing IPV. Moreover, given the results of the Wilcoxon–Mann–Whitney U test, we found that the IPV experiences group was significantly more likely to anticipate needing more help and cooperation from family, friends, and neighbors than the non-IPV experiences group. Prior studies in disasters and IPV have noted the protective role of social support for those experiencing both disaster and IPV (Kaniasty, 2012; Voth Schrag et al., 2020).

Our survey asked respondents to indicate how much help they were likely to need from government and nongovernment resources. Of the subsample with no reported experiences of IPV, almost one fifth (19.5%) thought they would need much or very much help from these resources. This is contrasted with almost a third (30.8%) of the subsample who reported IPV experiences that indicated they thought they would need much or very much help from government and nongovernment agencies. Although the statistical test proved not significant, these differences are descriptive but informative. The difference in the two subsamples is telling in that those affected by IPV may be more likely to need help from government and nongovernment agencies. During the pandemic this help may refer to unemployment, rent or mortgage assistance, and nutrition assistance. Whatever help this form may take is an opportunity for government and nongovernment agencies to communicate with individuals affected by IPV about potential resources available to them, particularly in times of crisis, such as the pandemic. These other forms of assistance could serve as a site of connection to IPV-related resources through affective communication. Similar to how medical professionals ask their patients if, “they feel safe at home,” COVID-19 testing sites could partner with IPV organizations to incorporate screenings for IPV during COVID-19 testing (Anurudran et al., 2020) and provide information handouts and hang posters, so that the general public can be aware of IPV resources that are available.

In addition, greater efforts in public awareness campaigns should be expended to raise awareness about the increased risk for IPV and the service availability for IPV protection during a pandemic (Jarnecke & Flanagan, 2020). As our findings show, experiences of IPV might prompt a need for more communication resources from their families and potentially from government and nongovernment sources for support and information. This could be the case because of the strain of IPV increases the need for such resources or it could be the case that IPV survivors start with lower levels of resources. Our analysis cannot say definitely, but our findings do point toward the need for more communication resources for IPV-reporters. This is particularly important given that those affected by IPV are more likely to need help from their friends, families, and neighbors during the ongoing pandemic. Furthermore, as research has suggested in other disaster contexts (First et al., 2017; Lauve-Moon & Ferreira, 2017), by providing public awareness on resources to the broader community, community members, trusted friends, neighbors, and family members may be better able to connect those affected by IPV with resources, such as shelters, treatment intervention program, and therapeutic professionals (e.g., social workers, psychologists, etc.). Likewise, government agencies and nonprofits that address IPV as a normal function of their missions should be included in policy decisions related to coordinating IPV responses and resources in a pandemic, to ensure that affirmative steps are taken to protect the members of this vulnerable population during the pandemic.

### Limitations

It is necessary to note some important limitations of this discussion. First, there may be other factors for why a greater number of those in the IPV subsample expressed needing help from neighbors, family, and friends and government and nongovernment agencies than the non-IPV subsample. Second, we did not ask on the survey how likely they were to utilize these resources, only if they anticipated needing help and cooperation from these sources. More research (suggested below in the research agenda section) is necessary to better determine the relationships between IPV, disasters, and effective communication strategies to reduce IPV in disaster contexts. Third, this sample was overrepresented by White participants and women and had high levels of education and differs from more U.S. representative samples.

### A Communication Ecology of IPV Research Agenda for the COVID-19 Era

In the current study, we highlight key predictors of stress related to IPV during COVID-19 and explore the importance of communicating these risk factors to increase protective responses for individuals experiencing IPV. By understanding predictors of stress related to IPV, we can extrapolate how communication resources can be improved and implemented to promote protective behaviors and coping during disasters like the COVID-19 pandemic. However, more research is needed to inform COVID-19 communication ecology focused on IPV. Below we highlight additional areas for research focused on IPV communication ecologies during COVID-19.

First, future research should examine the protective role of online IPV interventions and digital services (e.g., video conferencing and digital communication platforms) during COVID-19. IPV health information and IPV-related services have been widely accessed through internet-based interventions and mobile phone applications during COVID-19. Communicating with IPV survivors through technology (e.g., chat, video, text) comes with benefits and risks (e.g., safety, loss of privacy, and confidentiality) and more research is needed to further understand these benefits and risks during pandemics.

Second, future research should examine the role of social media in IPV communication ecologies for providing social support for IPV survivors. While prior research has long examined the important role of social support for individuals experiencing IPV, more research is needed to understand benefits and risks of seeking support online during COVID-19. For example, social media could be a platform for receiving emotional and informational support for IPV survivors (Chu et al., 2020). However, social media could also induce nonsupportive and victim-blaming messages (Whiting et al., 2019) and more research is needed to further understand the risks and benefits of social media in IPV communication ecologies.

Finally, research on IPV and disaster/pandemics is often considered a homogeneous category, thus implicitly assumes that individuals experiencing IPV essentially share the same life experiences. However, in reality, various dimensions of social inequality (e.g., gender race and ethnicity, migration, class, physical ability) can intersect to create unique challenges for individuals experiencing IPV during a pandemic. Future research should examine various dimensions of social vulnerability and IPV risk from an intersectional framework to further inform what factors constrain or facilitate the inclusion of different IPV communication resources during COVID-19.

## Conclusion

This study adds to the scant literature on IPV, communication ecologies, and pandemics. We endeavored to identify key predictors related to stressors due to COVID-19 of IPV. Given findings from this study, governments must do more to communicate the multiple stressors resulting from the pandemic, such as rent and nutrition stress, which likely exacerbate IPV. In addition to strengthening communication strategies alerting the public to these additional stressors and their potential to add to relationship stress, governments must do more to alleviate these stresses in the form of rental and nutrition assistance. Doing so will most likely reduce the exacerbated prevalence of IPV. Last, we have identified key areas for future research to extend knowledge into communication ecologies, IPV, and disasters to help inform decision makers on how to best communicate risk factors and coping strategies.

## Notes

1 Models were run with a series of predictor variables including sociodemographic variables. These effects were not statistically significant. We included the model that was statistically significant with the best combination of predictors to explain the outcome, membership in the IPV-experiences group.
